# Supplementary material for: Drug repurposing for aging research using model organisms
Source: Aging Cell. 2017 Jun 16;16(5):1006–15. doi: 10.1111/acel.12626 (PMC5595691; doi:10.1111/acel.12626)
Supplement: Supplementary file 7 — Data S1 Zip‐Archive of all report cards. [file ACEL-16-1006-s007.zip › RC_LDA.pdf]

LDA

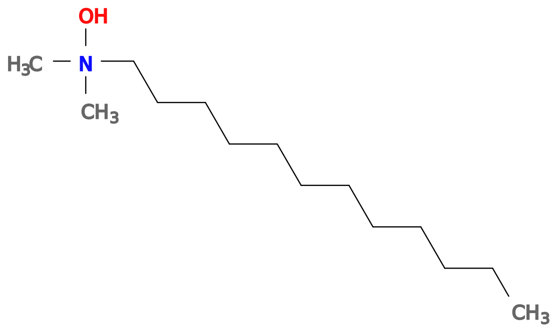

Database identifiers

ChEMBLCompound	CHEMBL1233973
DrugBank	DB04147
eMolecules	504917

Ranking

	Rank	Score
Drosophila	NA	NA
C. elegans	182/591	0.259

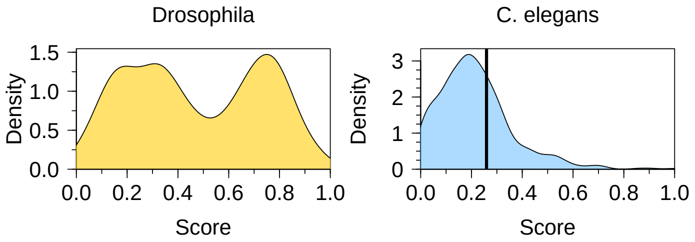

	Ageing implication	Domain conservation	Binding site conservation	Binding affinity	Bioavailability	Lipinski	Promiscuity	Purchasability	Drug approval	**Total**
Drosophila	NA	NA	NA	NA	NA	NA	NA	NA	NA	NA
C. elegans	1.0	0.982	1.0	0.169	0.51	0.0	-0.0	0.1	0.075	0.259

Names

- dodecyl-hydroxy-dimethyl-ammonium
- dodecyl-hydroxy-dimethyl-azanium
- hydroxy-lauryl-dimethyl-ammonium
- Lauryl Dimethylamine-N-Oxide

Roles

ChEBI entry None has no roles

Status

Approved drug (according to ChEMBL)	No
Classification (according to DrugBank)	experimental
Number of Rule of 5 violations	0
Binding affinity to original target in log units (RF-Score prediction)	3.41
Burns *C. elegans* bioavailability prediction	2.97

Compound Target Characteristics

Protein skinhead-1

Best gene implication in ageing for this target family came from gene P34707 annotated in UniProt release 2014_02. Annotation GO 8340 (determination of adult lifespan) was Inferred from Mutant Phenotype

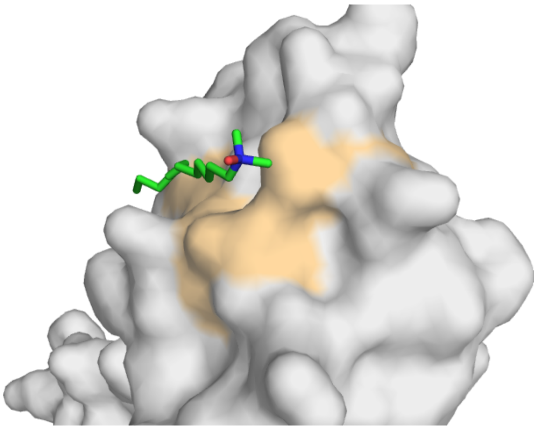

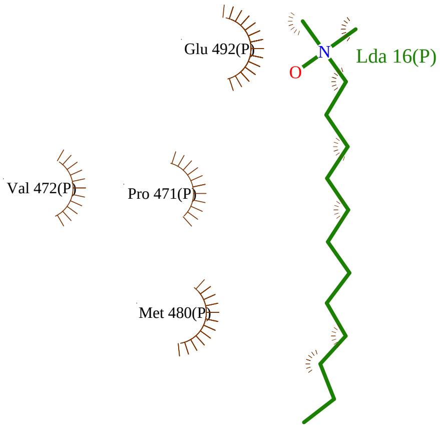

protein	amino acids contacts (binding site)
PDB:1skn:chainP:P34707	P V M E
sp:P34707:SKN1_CAEEL	P V M E

	whole protein		domain-based		contact-based	
protein	ident	simil	ident	simil	ident	simil
PDB:1skn:chainP:P34707	1.0	1.0	1.0	1.0	1.0	1.0
sp:P34707:SKN1_CAEEL	1.0	1.0	1.0	1.0	1.0	1.0

skn-1 (WBGene00004804) associated phenotypes

AB lineage variant, MS lineage variant, cell fate specification variant, cell fate transformation, drug induced life span variant, drug response variant, excess hypodermal cells, gastrulation variant, gene expression level reduced, gene expression variant, intestinal cell morphology variant, larval lethal, lethal, mRNA levels reduced, maternal effect lethal emb, muscle excess, no Intestine, no pharynx, organism oxidative stress response hypersensitive, pattern of gene expression variant, pattern of transgene expression variant, sterile, transgene expression undetectable

(Information from WormBase)

skn-1 (UniProt:P34707) annotation

Function: Required to specify the fate of ventral blastomeres in the early embryo, and postembryonically for the development of the intestine. Directly regulates expression of zygotically expressed med-1 and med-2 to direct mesendoderm development (PubMed:1547503, PubMed:11463373). Required for stl-1 mRNA up-regulation in response to oxidative stress and anoxia. In neurons, involved in mitochondrial fusion and behavioral recovery during reoxygenation (PubMed:24385935). (PubMed:11463373, PubMed:1547503, PubMed:24385935).
Subunit: Monomer. (PubMed:7939715).
Subcellular location: Nucleus (PubMed:7939715).

Tissue specificity: Postembryonic intestinal cells. (PubMed:1547503).
Developmental stage: Expressed both maternally and zygotically. (PubMed:1547503).
Domain: Alternative DNA-binding strategy due to lack of a leucine zipper dimerization segment: DNA binding domain binds to its cognate half-site, an N-terminal arm recognizes adjacent 5' AT-rich sequences in the minor groove and the intervening residues stabilize interactions of these two subdomains with DNA. (PubMed:9628487).

(Information from UniProt)
